# Vaccination of Health Care Workers to Protect Patients at Increased Risk for Acute Respiratory Disease

**DOI:** 10.3201/eid1808.111355

**Published:** 2012-08

**Authors:** Gayle P. Dolan, Rebecca C. Harris, Mandy Clarkson, Rachel Sokal, Gemma Morgan, Mitsuru Mukaigawara, Hiroshi Horiuchi, Rachel Hale, Laura Stormont, Laura Béchard-Evans, Yi-Sheng Chao, Sergey Eremin, Sara Martins, John S. Tam, Javier Peñalver, Arina Zanuzdana, Jonathan S. Nguyen-Van-Tam

**Affiliations:** University of Nottingham, Nottingham, UK (G.P. Dolan, R. Hale, J.S. Nguyen-Van-Tam);; World Health Organization, Geneva, Switzerland (R.C. Harris, M. Mukaigawara, L. Stormont, Laura Béchard-Evans, Y.-S. Chao, S. Eremin, S. Martins, J.S. Tam, J. Peñalver);; National Health Service Derbyshire County, Chesterfield, UK (M. Clarkson, R. Sokal);; Health Protection Agency South West, Gloucester, UK (G. Morgan);; Tokyo Medical Dental University, Tokyo, Japan (H. Horiuchi);; and University of Bielefeld, Bielefeld, Germany (A. Zanuzadana)

**Keywords:** vaccination, medical staff, influenza, transmission, patients, review, systematic, public health, viruses, health care workers, respiratory infections

## Abstract

Evidence is limited but sufficient to sustain current vaccination recommendations.

## Medscape CME Activity

Medscape, LLC is pleased to provide online continuing medical education (CME) for this journal article, allowing clinicians the opportunity to earn CME credit.

This activity has been planned and implemented in accordance with the Essential Areas and policies of the Accreditation Council for Continuing Medical Education through the joint sponsorship of Medscape, LLC and Emerging Infectious Diseases. Medscape, LLC is accredited by the ACCME to provide continuing medical education for physicians.

Medscape, LLC designates this Journal-based CME activity for a maximum of 1 *AMA PRA Category 1 Credit(s)*^TM^. Physicians should claim only the credit commensurate with the extent of their participation in the activity.

All other clinicians completing this activity will be issued a certificate of participation. To participate in this journal CME activity: (1) review the learning objectives and author disclosures; (2) study the education content; (3) take the post-test with a 70% minimum passing score and complete the evaluation at www.medscape.org/journal/eid; (4) view/print certificate.

**Release date: July 20, 2012; Expiration date: July 20, 2013**

## Learning Objectives

Upon completion of this activity, participants will be able to:

- Assess the impact of influenza infection among health care workers
- Analyze the methodology of research into vaccination of health care workers
- Evaluate the effects of health care worker vaccination on rates of influenza infection among patients
- Distinguish other patient-related outcomes of health care worker vaccination programs

## CME Editor

**Karen L. Foster,** Technical Writer/Editor, *Emerging Infectious Diseases. Disclosure: Karen L. Foster has disclosed no relevant financial relationships.*

## CME Author

**Charles P. Vega, MD,** Health Sciences Clinical Professor; Residency Director, Department of Family Medicine, University of California, Irvine. *Disclosure: Charles P. Vega, MD, has disclosed no relevant financial relationships.*

## Authors

*Disclosures:* ***Gayle P. Dolan, MBChB; Mandy Clarkson; Rachel Sokal; Gemma Morgan; Mitsuru Mukaigawara; Hiroshi Horiuchi, DDS, PhD; Rachel Hale; Laura Stormont; Laura Béchard-Evans; Sergey Eremin, MD, PhD; Sara Martins; John S. Tam; Javier Peñalver, MD;*** *and* ***Arina Zanuzadana*** *have disclosed no relevant financial relationships.* ***Rebecca C. Harris, MD,*** *has disclosed the following relevant financial relationships: served as an advisor or consultant for GlaxoSmithKline Biologicals, which began after major contributions to manuscript.* ***Yi-Sheng Chao*** *has disclosed the following relevant financial relationships: served as a consultant for Gere Biotechnology Ltd., Co., to review biomedical studies.* ***Jonathan S. Nguyen-Van-Tam, MD, PhD,*** *has disclosed the following relevant financial relationships: served as an advisor or consultant for F. Hoffman-LaRoche, Baxter AG, GlaxoSmithKline, and AstraZeneca, for which out of pocket travel expenses were reimbursed; currently in receipt of research funding from F. Hoffmann-La Roche, GlaxoSmithKline, and AstraZeneca.*
