# Supplementary material for: Vaccination of Health Care Workers to Protect Patients at Increased Risk for Acute Respiratory Disease
Source: Emerg Infect Dis. 2012 Aug;18(8):1225–34. doi: 10.3201/eid1808.111355 (PMC3414018; doi:10.3201/eid1808.111355)
Supplement: Related texts

# Review article: Vaccination of Health Care Workers to Protect Patients at Increased Risk for Acute Respiratory Disease

**DOI:** 10.3201/eid1808.111355

**Authors:** Gayle P. Dolan, Rebecca C. Harris, Mandy Clarkson, Rachel Sokal, Gemma Morgan, Mitsuru Mukaigawara, Hiroshi Horiuchi, Rachel Hale, Laura Stormont, Laura Béchard-Evans, Yi-Sheng Chao, Sergey Eremin, Sara Martins, John S. Tam, Javier Peñalver, Arina Zanuzadana, Jonathan S. Nguyen-Van-Tam

**Affiliations:** University of Nottingham, Nottingham, UK (G.P. Dolan, R. Hale, J.S. Nguyen-Van-Tam);; World Health Organization, Geneva, Switzerland (R.C. Harris, M. Mukaigawara, L. Stormont, Laura Béchard-Evans, Y.-S. Chao, S. Eremin, S. Martins, J.S. Tam, J. Peñalver);; National Health Service Derbyshire County, Chesterfield, UK (M. Clarkson, R. Sokal);; Health Protection Agency South West, Gloucester, UK (G. Morgan);; Tokyo Medical Dental University, Tokyo, Japan (H. Horiuchi);; and University of Bielefeld, Bielefeld, Germany (A. Zanuzadana)

## Abstract

Health care workers (HCWs) may transmit respiratory infection to patients. We assessed evidence for the effectiveness of vaccinating HCWs to provide indirect protection for patients at risk for severe or complicated disease after acute respiratory infection. We searched electronic health care databases and sources of gray literature by using a predefined strategy. Risk for bias was assessed by using validated tools, and results were synthesized by using a narrative approach. Seventeen of the 12,352 identified citations met the full inclusion criteria, and 3 additional articles were identified from reference or citation tracking. All considered influenza vaccination of HCWs, and most were conducted in long-term residential care settings. Consistency in the direction of effect was observed across several different outcome measures, suggesting a likely protective effect for patients in residential care settings. However, evidence was insufficient for us to confidently extrapolate this to other at-risk patient groups.

Respiratory disease is a leading cause of deaths worldwide, and influenza and pneumococcal infections are major contributors. Certain groups, such as persons >65 years of age or with chronic underlying health problems (*1*) are particularly vulnerable to severe respiratory disease and have poorer outcomes after infection than does the general population. These persons are likely to be frequent users of health care facilities, and outbreaks have been described in a range of high-risk environments, including acute care (*2**,**3*), pulmonary (*4*), and infectious diseases wards (*5*); organ transplant departments (*6*); children’s wards (*7**,**8*); neonatal intensive care units (*9*); and nursing homes (*10**,**11*). Severe respiratory infections often occur despite high vaccine coverage rates among patients, suggesting that seroconversion is suboptimal (*10*). Although the origin of infection often is difficult to establish, evidence from some outbreaks (*5**,**7**,**10**–**14*) suggests that transmission from HCWs to patients is likely.

 It is estimated from previous influenza seasons that ≈20% of HCWs have evidence of infection (*15*), although not necessarily acquired in the workplace. Young healthy adults often have asymptomatic infection, and ≈28%–59% might experience subclinical infection (*15*). Many persons with mild or subclinical illness continue to work while infectious, and even when illness is recognized, virus might be shed before symptom onset. In a randomized controlled trial among health care professionals, Wilde et al. demonstrated that influenza vaccine was 88% efficacious for reducing serologically confirmed influenza A infection and 89% efficacious for reducing serologically confirmed influenza B infection (*16*). Therefore, vaccination of HCWs has been widely recommended to provide direct protection for themselves and indirect protection for their patients (*1**,**17*).

Despite efforts to encourage influenza vaccination of HCWs, coverage has been historically poor. Recently, ethical arguments for mandatory influenza vaccination have been raised that focus not only on the direct and indirect benefits to staff and patient health but also on the economic consequences. Burls et al. (*18*) suggested that at a cost of £51–£405 (US$85–$675) per life-year saved, mandatory vaccination is likely to be cost-effective. However, evidence for the effectiveness of vaccinating HCWs for protecting vulnerable patients is limited.

Two recent systematic reviews considered the evidence for indirect protection of vulnerable patient groups after staff influenza vaccination (*18**,**19*). They suggest that vaccination of HCWs might be effective for reducing death and influenza-like illness (ILI) among elderly residents, but we are unaware of comparable data related to other at-risk groups. We aimed to identify and assess further evidence for the effect of vaccinating HCWs on patient groups most vulnerable to severe or complicated respiratory illness.

## Methods

The full study protocol is registered with the UK National Institute for Health Research International Prospective Register of Systematic Reviews (www.crd.york.ac.uk/PROSPERO [registration no. CRD420111092]). We searched several electronic health care databases, sources of evidence-based reviews, guidelines, and gray literature in accordance with the specifications of each database (Figure). In addition, we contacted domain experts and vaccine manufacturers to identify unpublished data and undertook citation and reference tracking for all included papers. Thesaurus-indexed and free text terms were defined for the population, intervention, and outcome parameters; peer reviewed; and adapted as necessary for each search engine.

**Figure:** Study selection for a review of the vaccination of health care workers to protect patients at risk for acute respiratory disease.

Eligibility criteria were defined a priori as follows:

- Types of study: any experiment, observational study, or systematic review reporting on the effectiveness of vaccination (including influenza or pneumococcal vaccines) of HCWs for protecting patients at higher risk for severe or complicated respiratory infection.
- Types of participants: persons at higher risk for severe or complicated illness as a result of acute respiratory infection (as defined in World Health Organization [*1*] and Advisory Committee on Immunization Practices guidance [*17*]), who have received or are receiving care from an HCW.
- Types of intervention: influenza or pneumococcal vaccination of any worker providing medical, nursing, social, or personal health care (because no uniformly accepted definition of an HCW exists, it was defined by the peer-reviewed terms specified in the search strategy).
- Types of outcome measure: cases or consultations, death or hospitalization for acute respiratory disease, influenza, ILI, or pneumococcal disease.

Published and unpublished reports from any year that were written in Chinese, English, French, Japanese, Portuguese, Russian, or Spanish were considered. A 3-stage process was used to assess eligibility for inclusion screening first by title, then abstract, and then full text. Two reviewers undertook this in parallel for stages 1 and 2 and independently for stage 3. Consensus was reached by discussion; when reviewers disagreed, a third reviewer was consulted for a final decision. Where multiple reports were identified for the same piece of original research, the most recent peer-reviewed source was selected.

Two reviewers independently extracted data from each included, by using a predefined, piloted template. The risk for bias was assessed by using the Cochrane Collaboration tool (*20*) for experimental and prospective cohort studies, the Downs and Black tool (*21*) for other observational studies, and the US Agency for Healthcare Research and Quality (*22*) domain and element-based evaluation instrument for systematic reviews. Again, consensus was reached by discussion, with engagement of a third reviewer as necessary. No additional information was sought from corresponding authors. Data were synthesized qualitatively by using a narrative approach in accordance with the framework described by the Economic and Social Research Council and recommended by the University of York Centre for Reviews and Dissemination (*23*).

## Results

### Study Selection

We identified 12,352 citations (Figure): 10,713 from health care databases and the remainder from additional sources. Seventeen studies met the inclusion criteria at the full text stage; 3 others were identified from citation or reference tracking. Of these, 14 were primary research articles; 4 were cluster randomized controlled trials (RCTs), and 10 were observational studies. Four of the remaining 6 articles were different versions of a report relating to 1 systematic review, and the other 2 were different versions of a report relating to a second systematic review. One of these systematic reviews (*18*) provided a qualitative analysis of 2 of the earliest cluster RCTs (*24**,**25*), and the other (*19*) provided a quantitative meta-analysis of all 4 cluster RCTs (*24**–**27*) and 1 additional observational study (*28*). We used the most recent and detailed version of each review published in a peer-reviewed source in this study.

All of the primary studies considered influenza vaccination of HCWs (Table A1); therefore, we discarded our planned subanalysis relating to pneumococcal vaccination. Only 4 studies (*24**–**26**,**29*) defined HCW, even though this definition is likely to affect the probability of transmission and therefore the magnitude of observed effects. Where reported, vaccination among staff ranged from ≈35% to 70% in the intervention arm and from none to 32% in the control arm of experimental studies and from 12% to 90% in observational studies. Eleven of the primary research studies were conducted in long-term care facilities; the remainder were conducted in renal dialysis facilities (*30*), a pediatric hospital (*31*), and an adult oncology hospital (*32*) (1 study each). Where reported, vaccination coverage among patient populations ranged from 0% to ≈90%, and few studies considered additional infection control practices, such as hand washing, duration of contact, or use of face masks, which vary and again influence the propensity for transmission.

### Risk for Bias

#### Cochrane Collaboration Tool

Concerns arose largely from the lack of blinding of participants or study personnel (Table 1). Although the effect was likely to be minimal with regard to the primary outcome for all 4 RCTs (all-cause mortality), it might have resulted in underestimation or overestimation of additional, more subjective, outcome measures, such as incidence of ILI.

**Table 1:** Risk for bias assessed by using the Cochrane Collaboration tool in a review of the vaccination of health care workers to protect patients at risk for acute respiratory disease

Study	Sequence generation	Allocation concealment	Blinding of participants, personnel and outcome assessors			Incomplete outcome data		Selective outcome reporting	Other sources of bias
			Primary outcome	Secondary outcomes		Primary outcome	Secondary outcomes		
Lemaitre et al. (*26*)									
Hayward et al. (*27*)									
Carman et al. (*24*)									
Potter et al. (*25*)									
Saito et al. (*33*)									
Oshitani et al. (*28*)									

*Black shading, low risk of bias; light gray shading, uncertain risk of bias; dark gray shading, high risk of bias; blank cells, no secondary outcome measure reported.

All studies, except for that by Lemaitre et al. (*26*), were judged to be at some further risk for bias. This included selection bias (inadequate description of selection criteria [*24**,**25**,**33*] or sequence allocation [*25**,**28**,**33*]), performance bias (lack of detail about allocation concealment [*25**,**26*]), and measurement bias (no clearly defined outcome measure [*28*]).

#### Downs and Black Tool

The Downs and Black tool (Table 2) considers 5 assessment domains, but because most observational studies identified were primarily descriptive, we excluded the power domain in this review. Scores ranged from 3/27 (*34*) to 10/27 (*29**,**30**,**35*), with higher scores representing lower risk for bias. None of the studies provided sufficient detail about the patient population, and only 1 (*29*) described principal confounders. Other concerns about reporting related to lack of detail of study objectives (*29**,**32**,**34*), a priori definition of outcome measures (*32**,**34**–**37*) or those lost to follow up (*35*), failure to provide sufficient detail of statistical analysis (*29**,**30**,**34**–**37*), lack of randomization or blinding, and failure to adjust outcome measures.

**Table 2:** Risk for bias by using the Downs and Black tool in a review of the vaccination of health care workers to protect patients at risk for acute respiratory disease

Study	Type of score (maximum score)*				
	Reporting (11)	External validity (3)	Internal validity, bias (7)	Internal validity, confounding (6)	Total (27)
Ando et al. (*30*)	5	2	2	1	10
Shugarman et al. (*35*)	6	0	1	3	10
Kanaoka et al. (*29*)	5	1	3	1	10
Monto et al. (*36*)	5	0	2	2	9
Weinstock et al. (*32*)	4	0	4	1	9
Stevenson et al. (*37*)	4	1	2	1	8
Munford et al. (*34*)	2	0	0	1	3

*Maximum score indicates lowest risk of bias for each domain.

#### Agency for Healthcare Research and Quality Tool

We assessed the 2 identified systematic reviews (*18**,**19*) by using the Agency for Healthcare Research and Quality tool (*22*). Both appeared to be at a comparatively low risk for bias, providing a clearly defined research question, search strategy, inclusion and exclusion criteria, and description of outcomes. However, details were lacking about blinding of reviewers to authorship and measurement of agreement in extracting data, which might have resulted in measurement bias.

### Synthesis of Results

#### Cases or Consultations for Acute Respiratory Disease

One RCT reported data (*25*) for 2 measures of consultation for respiratory disease; episodes of lower respiratory tract infection and suspected viral illness (Table 3). In addition, the estimate for lower respiratory tract infection was adjusted for clustering by Thomas et al. (*19*). Both measures demonstrated reduced odds, and results were significant for suspected viral illness when vaccinated and nonvaccinated patients were considered together.

**Table 3:** Cases of and consultations for acute respiratory disease in a review of the vaccination of health care workers to protect patients at risk for acute respiratory disease*

Outcome measure (study)	Study design	Method of assessment	Measure of effect in patient population	Effect estimate (95% CI)
Clinically defined episodes of viral illness (Potter et al. [*25*])	Cluster RCT	Not defined. No. episodes recorded by study nurses.	OR, nonvaccinated and vaccinated patients	**0.64 (0.48–0.87)**
			OR, vaccinated patients	**0.40 (0.26–0.62)**
			OR, nonvaccinated patients	0.98 (0.65–1.48)
Lower respiratory tract infection				
Potter et al. (*25*)	Cluster RCT	Defined as 1) pulmonary crackles, wheeze, or tachypnea plus temperature >37.0°C or leukocyte count >10 × 10^9^/L or 2) a positive sputum culture. No. episodes recorded by study nurses.	OR, nonvaccinated and vaccinated patients	0.69 (0.40–1.19)
			OR, vaccinated patients	0.59 (0.25–1.38)
			OR, nonvaccinated patients	0.77 (0.38–1.57)
Thomas et al. (*19*)	Pooled data		OR, adjusted for clustering	0.71 (0.29–1.71)†

*RCT, randomized controlled trial; OR, odds ratio. **Boldface** indicates statistical significance. †p = 0.44. p value not reported for other categories.

The study by Potter et al. (*25*) was considered to be at a higher risk for bias than the other RCTs identified; thus, the strength of evidence for these outcomes is questionable. In addition, the measures considered are nonspecific, and the observed effects cannot necessarily be attributed to reduced influenza infection. Nasopharyngeal samples were taken from a subset of patients within 48 hours after symptoms developed; no samples were positive for influenza on immunofluorescence assay.

#### Cases or Consultations for Influenza or ILI

Data were reported in 13 studies for 5 outcome measures of influenza/ILI. Eight primary studies measured clinically defined influenza/ILI (Table A2, Table 4).

**Table 4:** Clinically defined outbreaks and clusters of ILI in a review of the vaccination of health care workers to protect patients at risk for acute respiratory disease*

Study	Study design	Method of assessment	Measure of effect in patient population	Effect estimate (95% CI)
Oshitani et al. (*28*)	Prospective cohort	Defined as ILI >10% of total resident population. Mandatory reporting by survey.	OR, unadjusted facilities with >10 staff members vaccinated vs. those with <10 staff members vaccinated	**0.30 (0.09–0.69)†**
Stevenson et al. (*37*)	Cross-sectional	No definition provided. Reporting by survey.	χ**^2^** test for trend; logistic regression	χ^2^ p = 0.03; logistic regression p = 0.08
Shugarman et al. (*35*)	Cross-sectional	Defined as >3 residents within a 72-h period with influenza-like symptoms, sudden onset of fever, or “feverishness” and >1 of the following respiratory symptoms; sore throat, runny nose, cough, or nasal congestion. Reporting by survey.	OR, facilities with staff vaccination coverage >55% and patient vaccination coverage >89%, vs. those with lower coverage	**0.39 (0.17–0.87)†**

*ILI, influenza-like illness; OR, odds ratio. **Boldface** indicates statistical significance. †p value not reported.

Three RCTs (*25**–**27*) measured cases of ILI, and these data were pooled by Thomas et al. (*19*) to demonstrate a statistically significant reduction in odds. Two observational studies (*28**,**33*) also measured cases of clinically defined ILI, demonstrating statistically significant reductions in risk, although the threshold of staff vaccination coverage used to categorize facilities in these studies varied (Oshitani [*28*] considering facilities where more or fewer than 10 staff were vaccinated, and Saito [*33*] comparing facilities with <40%, 40%–59%, and >60% coverage among staff). A third observational study (*29*) reported no correlation between staff vaccination coverage and cases of influenza in patients, although the relative change in vaccination coverage (79%–91%) was small and thus any difference in the number of cases was probably difficult to detect. The magnitude of reported effects varied, most notably by influenza season in the study of Hayward et al. (*27*), and with patient vaccination status in the study of Potter et al. (*25*).

One study measured general practitioners consultations for ILI (*27*). An inconsistent effect was demonstrated across different periods of influenza activity, but pooled data suggested an overall statistically significant reduction in the odds of consultation after vaccination of HCWs.

Three observational studies (*28**,**35**,**37*) demonstrated a statistically significant protective effect of staff vaccination against clinically defined outbreaks of ILI in patients (Table 4). The thresholds used to categorize facilities on the basis of staff vaccination coverage again varied among studies, and these data were considered to be at relatively high risk for bias.

Measures of laboratory-confirmed infection (Table A3) were less frequently reported and generally based on small samples of data at high risk for bias. Five studies measured laboratory-diagnosed influenza (*24**,**25**,**31**,**32**,**36*), although 1 reported no statistical analysis (*25*). Different methods of defining laboratory confirmation were used (Table A3). Thomas et al. (*19*) pooled data from the 2 RCTs (*24**,**25*) to demonstrate a small nonsignificant protective effect. This result is supported by evidence from 2 additional observational studies (*31**,**32*), which indicated a statistically significant reduction in the proportion of laboratory-confirmed cases of nosocomial influenza among inpatient pediatric and oncology patients after implementation of vaccination campaigns. In addition, Monto et al. (*36*) measured outbreaks of laboratory-diagnosed influenza, and this was the only study not to demonstrate a protective effect of vaccinating HCWs. The authors reported a higher, but nonsignificant, median vaccination coverage among staff in homes experiencing outbreaks.

#### Deaths from Respiratory Infection, ILI, or Acute or Respiratory Disease or Its Complications

Evidence for 5 measures of death was identified (Table 5). All 4 RCTs (*24**–**27*) considered all-cause death as their primary objective, providing the strongest evidence on the basis of study design. Although not defined a priori as an outcome of interest for this review, data were therefore extracted. These were pooled by Thomas et al. (*19*) to demonstrate a statistically significant protective effect.

**Table 5:** Measures of death in a review of the vaccination of health care workers to protect patients at risk for acute respiratory disease*

Outcome measure and study	Study design	Method of assessment	Measure of effect in patient population	Effect estimate (95% CI), p value
All-cause mortality				
Potter et al. (*25*)	Cluster RCT	Death certificate	OR, vaccinated and nonvaccinated patients	**0.56 (0.40–0.80)†**
			OR, vaccinated patients	**0.57 (0.35–0.91)†**
			OR, nonvaccinated patients	**0.56 (0.34–0.94)†**
Carman et al. (*24*)	Cluster RCT	Not stated	OR	0.62 (0.36–1.04), p = 0.092
Hayward et al. (*27*)	Cluster RCT	Reporting by lead nurse	Rate difference, epidemic period 1	**–0.05 (–0.07 to –0.02)**, p = 0.002
			Rate difference, epidemic period 2	–0.01 (–0.04 to 0.02), p = 0.49
			Rate difference, nonepidemic period 1	0.00 (–0.03 to 0.03), p = 0.93
			Rate difference, nonepidemic period 2	0.01 (–0.03 to 0.04), p = 0.70
Lemaitre et al. (*26*)	Cluster RCT	Not stated	OR	0.86 (0.72–1.02), p = 0.08
Thomas et al. (*19*)	Pooled data		OR, adjusted for clustering	**0.68 (0.55–0.84),** p<0.001
Respiratory deaths: Lemaitre et al. (*26*)	Cluster RCT	Reporting by study nurses	OR	1.55 (0.59–4.10), p = 0.38
Pneumonia-associated deaths				
Potter et al. (*25*)	Cluster RCT	Reporting by lead nurse	OR, vaccinated and nonvaccinated patients	**0.60 (0.37–0.97)†**
			OR, vaccinated patients	0.56 (0.28–1.13)†
			OR, nonvaccinated patients	0.64 (0.33–1.23)†
Thomas et al. (*19*)	Pooled data		Risk ratio, adjusted for clustering	0.87 (0.47**–**1.64), p = 0.67
Death with influenza-like illness				
Hayward et al. (*27*)	Cluster RCT	Reporting by lead nurse	Rate difference, epidemic period 1	–0.01 (–0.02 to 0.01), p = 0.24
			Rate difference, epidemic period 2	–0.01 (–0.03 to 0.00), p = 0.08
			Rate difference, nonepidemic period 1	–0.01 (–0.04 to 0.02), p = 0.59
			Rate difference, nonepidemic period 2	0.01 (–0.01 to 0.02), p = 0.35
Thomas et al. (*19*)	Pooled data		OR, adjusted for clustering	0.72 (0.31–1.70), p = 0.45
Laboratory-diagnosed influenza at death: Carman et al. (*24*)	Cluster RCT	Nasal swab within 12 h before death	Difference in proportions, influenza positive at death	20%, p = 0.055

*RCT, randomized controlled trial; OR, odds ratio. **Boldface** indicates statistical significance. Shaded fields represent pooled data. †p value not reported.

Although at higher risk for bias, supporting data were provided for 4 more-specific measures. Thomas et al. (*19*) pooled data from 2 RCTs, 1 measuring deaths after pneumonia (*25*), the other measuring respiratory deaths (*26*), and demonstrated a small nonsignificant protective effect. However, the validity of this pooled analysis was questionable because how these outcomes were defined was not clear. Nonsignificant reductions in risk also were observed for laboratory-diagnosed influenza at death (*24*) and death after ILI (*27*). Again, the direction of the observed effects was largely consistent with other measures, providing further support for a hypothesis of indirect protection.

#### Admission to a Health Care Facility or Any Other Suggestion of Impact

Hospitalization was measured in 2 RCTs (*26**,**27*), pooled data suggesting a small, nonsignificant effect (Table 6). One RCT also measured hospitalization for respiratory causes (*26*) and 1 admission to hospital with ILI (*27*), although neither demonstrated any apparent effect. This result is particularly noteworthy given the observed decrease in deaths and might reflect health-seeking behaviors.

**Table 6:** Measures of hospitalization in a review of the vaccination of health care workers to protect patients at risk for acute respiratory disease*

Outcome measure and study	Study design	Method of assessment	Measure of effect in patient population	Effect estimate (95% CI)
Hospitalization				
Hayward et al. (*27*)	Cluster RCT	Reporting by lead nurse	Rate difference, epidemic period 1	−0.02 (−0.05 to 0.02), p = 0.35
			Rate difference, epidemic period 2	0.00 (−0.03 to 0.04), p = 0.84
			Rate difference, nonepidemic period 1	0.00 (−0.04 to 0.03), p = 0.80
			Rate difference, nonepidemic period 2	0.00 (−0.03 to 0.03), p = 0.86
Lemaitre et al. (*26*)	Cluster RCT	Not stated	OR	1.03 (0.76–1.40), p = 0.85
Thomas et al. (*19*)	Pooled data		OR, adjusted for clustering	0.90 (0.66 to 1.21), p = 0.47
Hospitalization for respiratory causes: Lemaitre et al. (*26*)	Cluster RCT	Not stated	OR	1.01 (0.43–2.34), p = 0.98
Admissions to hospital with influenza-like illness: Hayward et al. (*27*)	Cluster RCT	Reporting by lead nurse	Rate difference, epidemic period 1	−0.02 (−0.03 to 0.00), p = 0.009
			Rate difference, epidemic period 2	0.00 (−0.02 to 0.02), p = 0.99
			Rate difference, nonepidemic period 1	−0.01 (−0.02 to 0.01), p = 0.32
			Rate difference, nonepidemic period 2	0.01 (0.00–0.02), p = 0.31

*RCT, randomized controlled trial; OR, odds ratio. **Boldface** indicates statistical significance. Shading indicates pooled data.

## Discussion

Evidence is limited for the effectiveness of vaccination of HCWs for protecting patients at higher risk for severe or complicated respiratory illness. Despite the broad question posed, extensive searching, and large number of resultant hits, our search resulted in a low yield of studies, all of which focused on influenza with no consideration for pneumococcal infection. This finding is perhaps not surprising because pneumococcal vaccination is not routinely recommended for HCWs and little, if any, evidence exists of nosocomial spread. A consistent direction of effect was observed across multiple outcome measures, with virtually all studies noting a trend toward a protective effect of vaccinating HCWs. This consistency adds to the degree of confidence in interpreting our overall findings. Given that most studies were carried out in long-term care facilities, we conclude that vaccination of HCWs against influenza is likely to offer protection for this patient group. However, future reviews that specifically examine the effect of vaccinating other outpatient providers, such as home HCWs and hospital staff in acute care, short-stay settings, would clearly be of value. These findings are more difficult to extrapolate to other at-risk groups, although some, albeit limited, evidence was identified from other settings to suggest a similar effect.

The results of all 4 RCTs (*24**–**27*) and 1 of the observational studies identified (*28*) previously had been pooled in a quantitative meta-analysis (*19*). The authors of this analysis concluded that evidence is lacking that vaccinating HCWs prevents influenza infection in elderly patients because the apparent benefits were confined to nonspecific outcome measures. We considered additional observational data that demonstrate consistency in the direction of the observed effects across specific and nonspecific outcome measures. Although the strength of evidence for more-specific measures is generally much weaker, these findings add greater weight to the hypothesis of a potential protective effect.

The recent position statement by the Society for Healthcare Epidemiology of America (*38*) suggests that further studies are not needed because the biological rationale for vaccination does not vary by practice setting. However, effect size might vary considerably because of patient characteristics and care patterns (staff deployment and duration of inpatient stay), and further evidence is needed among the most at-risk groups where benefits are probably greatest, to enable prioritization of resources, particularly where vaccine shortages or resource limitations might exist.

Previous authors have suggested that vaccination of HCWs might enable development of herd immunity. Realistically, herd immunity is difficult to achieve in health care settings, especially acute care short-stay settings, because of patient admissions and discharges, visitors, and staff turnover. That said, herd immunity might not be necessary to benefit patients; modeling studies (*39*) suggest a direct association between coverage and attack rates. Such studies (*39*) also suggest variation in the potential for transmission of infection by different staff groups, which should be explored in further detail.

This field of research has some inherent problems. These difficulties result in part from the difficulty of isolating the effect of HCW vaccination, disentangling it from other factors that might influence patient outcomes, such as patient vaccination (as demonstrated by Potter et al. [*25*]) and background influenza activity (as demonstrated by Hayward et al. [*27*]). Staff vaccination itself might be linked to additional confounding variables, such as organizational culture and professional beliefs. In fact, such confounding might explain the difference in findings between the work of Monto (*36*) and the other authors. Prospective collection of information relating to relevant transmission factors and infection control measures that were largely overlooked by the studies in this review should be used to enable appropriate adjustment in future studies. Furthermore, the most appropriate outcome measures are difficult to define because not all persons with laboratory-confirmed infection have symptoms of illness and vice versa. Future studies thus need to demonstrate consistent effects for a range of clearly defined outcomes by using valid measures across several different influenza seasons, with sufficient power to detect true underlying effects.

The findings of our review are subject to several limitations. Because 11 of the 14 primary research articles considered outcomes in long-term care facilities, generalizability to other at-risk groups is limited. In addition, we did not attempt to contact authors of original studies, and the conclusions drawn are limited by the reported detail. Although the number of reviewers was limited as far as possible, some inconsistency might have occurred in the selection, extraction, and assessment of data introducing potential bias, particularly where the opportunity for subjective judgment existed. We attempted to minimize inconsistency by using several standard assessment tools, but their use was limited by lack of information where components were not conducted because of the nature of the study design. Meta-analysis of the 4 RCTs identified had already been conducted, and although we identified additional observational data, the observed heterogeneity limited any further quantitative analysis.

Some wider possible effects of HCW vaccination, such as reduction in absenteeism because of illness, are beyond the scope of this review. Ethically, autonomy needs to be balanced with nonmaleficence, and this need must be addressed when policy decisions about vaccination are considered. Anikeeva et al. (*40*) reported that in a review of 15 studies focusing on the reasons staff accept influenza vaccine, self-protection was the most important. However, patient protection also was perceived as important, particularly among HCWs in settings with higher risk patients (*40*). Nevertheless, HCWs would be justified in claiming that the current evidence base is not especially strong and heavily weighted toward the benefits to patients receiving care in long-term care facilities, although limited evidence would not necessarily legitimize nonacceptance.

The existing evidence base is sufficient to sustain current recommendations for vaccinating HCWs on the grounds that some protection of high-risk patients against influenza seems likely. However, vaccination should be considered 1 element of a broad package of infection prevention and control measures, such as good hand and respiratory hygiene, environmental cleaning, protection against respiratory droplets, and cohorted care during outbreaks. Well-designed studies that strengthen the evidence base might increase compliance with guidelines, resulting in improved coverage.

## Appendix

**Table A1:** Characteristics of primary studies of the vaccination of HCWs to protect patients at risk for acute respiratory disease*

Study	Study design	Setting	Intervention/exposure	No. HCWs	No. patients	Primary outcome in patient population	Duration of follow-up (no. influenza seasons)
Carman et al., 2000 (*24*)	Cluster RCT	Geriatric hospitals, Scotland	Routine offer of vaccination or no offer	1,217 in intervention arm (620 vaccinated); not stated for control arm	749 in intervention arm; 688 in control arm	All-cause mortality	6 mo (1)
Potter et al., 1997 (*25*)	Cluster RCT	Geriatric hospitals, Scotland	Stratified by policy for patient vaccination; randomized to routine offer of influenza vaccine to HCWs or no vaccination of HCWs	1,078 in intervention arm (440 HCWs, (of which 67% vaccinated), in vaccinated patient group, and 638 HCWs, (of which 57% vaccinated), in nonvaccinated patient group); not stated for control arm	490 patients in HCW intervention arm (230 patients in vaccinated patient group and 260 in nonvaccinated patient group); 544 in control arm (283 in vaccinated patient group and 261 in nonvaccinated patient group)	All-cause mortality	5 mo (1)
Lemaitre et al., 2009 (*26*)	Cluster RCT	Nursing homes, France	Promotional influenza vaccination campaign or provision of routine information	989 in intervention arm (678 vaccinated); 1,015 in control arm	1,722 intervention arm; 1,678 control arm	Al-cause mortality	2.5 mo (1)
Hayward et al., 2006 (*27*)	Cluster RCT	Care homes, England	Staff influenza vaccination policy or policy of not actively promoting vaccination	Year 1: 1,610 in intervention arm (570 vaccinated); 1,674 in control arm. Year 2: 1,726 in intervention arm (517 vaccinated); 1,766 in control arm	Year 1: 1,233 intervention arm; 1,371 control arm. Year 2: 1,270 intervention arm; 1,391 control arm.	All-cause mortality	8 mo (2)
Oshitani et al., 2000 (*28*)	Prospective cohort	Nursing homes/ geriatric health service facilities, Japan	Staff and patient influenza vaccination	7,459 (1,532 vaccinated)	12,784 (3,933 vaccinated) residents in 149 facilities	ILI cases	3 mo (1)
Kanaoka et al., 2010 (*29*)	Cross-sectional study	Long-term care facility, Japan	Influenza vaccination of staff and patients	179–188 over 7-y period (vaccination coverage 79%–91%)	180–185 over 7-y period (vaccination coverage 45%–72%)	Influenza cases	Seven 6-mo periods (7)
Ando et al., 2010 (*30*)	Cross-sectional study	Hemodialysis clinics, Japan	Staff influenza vaccination coverage	691–1,221 over 6-mo period (vaccination coverage 45%–87%)	2,881–5,055 over 6-mo period (vaccination coverage not reported)	ILI cases	6 mo (1)
Engels et al., 2005 (*31*)	Ecologic	Pediatric hospital, unknown	Staff influenza vaccination	Not stated. Vaccination coverage reported as negligible in 1999–2002 (before implementation); 48% in 2002 and 47% in 2003 (after implementation).	Not stated	Laboratory-diagnosed influenza	Exact duration not clear (3)
Weinstock et al., 2000 (*32*)	Ecologic	Adult oncology hospital, United States	Staff influenza vaccination with concurrent promotional campaign	Not clear. 1,457 vaccinated before implementation 1997–98 (12% of bone marrow transplant HCWs) and 1,956 vaccinated after implementation in 1998–99 (58% of bone marrow transplant HCWs)	Not stated	Laboratory-confirmed influenza	7 mo (1)
Saito et al., 2002 (*33*)	Prospective cohort	Care home, Japan	Staff and patient influenza vaccination	Year 1: 440 (154 vaccinated). Year 2: 517 (360 vaccinated)	Year 1: 699 (331 vaccinated). Year 2: 930 (743 vaccinated)	ILI cases	8 mo (2)
Munford et al., 2008 (*34*)	Ecologic	Extended-care unit, Canada	Staff influenza vaccination with concurrent promotional campaign	Numbers unknown. Vaccination coverage was 39% in 2005–06 (before implementation) 84% in 2006–07 and 83% in 2007–08 (after implementation)	Not specified. Reported to be ≈150 residents in unit	ILI cases	Exact duration not clear (3)
Shugarman et al., 2006 (*35*)	Cross-sectional study	Not stated; assumed care homes, United States	Staff and patient influenza vaccination coverage	301 homes with 28,174 staff. Categorized as high uptake where >55% coverage and low where uptake <55%.	301 homes with 30,371 patients. Categorized as high uptake where >89% coverage, low where <89% coverage.	ILI clusters	8 mo (1)
Monto et al., 2004 (*36*)	Observational with case–control comparison	Nursing homes, United States	Staff influenza vaccination coverage	Not stated (mean vaccination coverage 32%)	Not stated (31 homes with mean of 136 residents and mean vaccination coverage 76%)	Laboratory-diagnosed influenza outbreaks	5 mo (1)
Stevenson et al., 2001 (*37*)	Cross-sectional study	Long-term elderly care facilities, Canada	Staff and patient influenza vaccination coverage	Not reported (1,270 facilities responded in 1991, 430 in 1995, and 380 in 1999)	Not reported (1,270 facilities responded in 1991, 430 in 1995, and 380 in 1999)	ILI outbreaks	(1)

*HCW, health care worker; RCT, randomized controlled trial; ILI, influenza-like illness.

**Table A2:** Cases and consultations for clinically diagnosed influenza and ILI in a review of the vaccination of HCWs to protect patients at risk for acute respiratory disease*

Outcome measure, study	Study design	Method of assessment	Measure of effect in patient population	Effect estimate (95% CI)
Clinically defined ILI				
Potter et al. (*25*)	Cluster RCT	Defined as temperature >37.0°C plus >1 of the following symptoms: new-onset cough, coryza, sore throat, malaise, headache or muscle pains. Reported by nurse.	OR, nonvaccinated and vaccinated patients	**0.57 (0.34–0.94)†**
			OR, vaccinated patients	**0.24 (0.10–0.59)†**
			OR, nonvaccinated patients	0.86 (0.46**–**1.61)†
Lemaitre et al. (*26*)	Cluster RCT	Defined as temperature >37.8°C plus onset or worsening of respiratory symptoms. Method of reporting not stated.	OR	**0.69 (0.52–0.91), p = 0.007**
Hayward et al. (*27*)	Cluster RCT	Defined as fever >37.8°C or deterioration, plus onset or worsening of respiratory symptoms. Reported by lead nurse.	Rate difference, epidemic period 1	**–0.09 (–0.14 to –0.03, p = 0.004**
			Rate difference, epidemic period 2	0.00 (**–**0.06 to 0.06, p = 0.93)
			Rate difference, nonepidemic period 1	0.00 (**–**0.05 to 0.05, p = 0.93)
			Rate difference, nonepidemic period 2	0.03 (**–**0.07 to 0.12, p = 0.57)
Thomas et al. (*19*)	Pooled data		Risk ratio, adjusted for clustering	**0.71 (0.58–0.88), p = 0.0018**
Saito et al. (*33*)	Prospective cohort	Defined as sudden onset of fever of >37.8°C for >1 d and >1 of the following signs and symptoms: cough, sore throat, coryza. Routine assessment by nursing staff.	RR, middle compared with low HCW vaccination rate, season 1	0.90 (0.49**–**1.65), p = 0.7
			RR, high compared with low HCW vaccination rate, season 1	**0.19 (0.10–0.36), p<0.001**
			RR, middle compared with low HCW vaccination rate, season 2	**0.36 (0.17–0.75), p<0.01**
			RR, high compared with low HCW vaccination rate. season 2	0.51 (0.25**–**1.04), p = 0.07
Oshitani et al. (*28*)	Prospective cohort	Definition not provided. Mandatory reporting by survey.	OR, exposure to vaccinated staff compared with unvaccinated staff	**0.28 (0.23–0.32)†**
Kanaoka et al. (*29*)	Cross-sectional	Defined as symptoms of ILI with a positive antigen test or symptoms of ILI with a negative antigen test but clinical diagnosis by multiple clinicians. Method of reporting not stated.	Spearman rank correlation, hospital personnel vaccination coverage and no. influenza cases	r = 0.379, p = 0.459
General practitioner consultations for ILI				
Hayward et al. (*27*)	Cluster RCT	Reporting by lead nurse	Rate difference, epidemic period 1	**–0.07 (–0.12 to –0.02), p = 0.002**
			Rate difference, epidemic period 2	**–**0.01 (**–**0.07 to 0.05), p = 0.77
			Rate difference, nonepidemic period 1	**–**0.01 (**–**0.06 to 0.041), p = 0.74
			Rate difference, nonepidemic period 2	0.00 (**–**0.08 to 0.08), p = 0.95
Thomas et al. (*19*)	Pooled data		OR, adjusted for clustering	**0.48 (0.33–0.69), p<0.001**

*ILI, influenza-like illness; HCW, health care worker; RCT, randomized controlled trial; OR, odds ratio; RR, relative risk. **Boldface** indicates statistical significance. Shaded fields represent pooled data. †p value not reported.

**Table A3:** Cases and consultation for laboratory-diagnosed influenza in a review of the vaccination of HCWs to protect patients at risk for acute respiratory disease*

Outcome measure and study	Study design	Method of assessment	Measure of effect in patient population	Effect estimate (95% CI)
Laboratory-diagnosed influenza				
Carman et al. (*24*)	Cluster RCT	Nasal and oropharyngeal swabs (tissue culture/RT-PCR)	Difference in proportions	Routine surveillance, p = 0.42; opportunistic sampling, p = 0.54
Thomas et al (*19*)	Pooled data†		OR, adjusted for clustering	0.87 (0.38–1.99), p = 0.74
Weinstock et al. (*32*)	Ecologic	Clinical definition plus positive shell viral assay, enzyme immunoassay, or tissue culture	Difference in proportions (before to after implementation)	**72.1% decrease, p<0.01**
Engels et al. (*31*)	Ecologic	Nasopharyngeal aspirate culture	Difference in proportions (before to after implementation)	**p<0.001**, all children); p = 0.05, children with underlying disease; **p<0.05**, children <24 mos
Laboratory-diagnosed outbreaks of ILI, Monto et al. (*36*)	Cohort with case–control analysis	Laboratory confirmation of cases by using rapid antigen detection testing, viral cell culture, or RT-PCR	Difference in proportions	Median staff vaccination coverage in homes with outbreaks = 42% vs. 24% in homes without outbreaks, p>0.05

*HCW, health care worker; RCT, randomized controlled trial; RT-PCR, reverse transcription PCR; OR, odds ratio. **Boldface** indicates statistical significance. Shading indicates pooled data. †Pooled by using data from Potter and Carman but no estimate of effect reported by Potter et al.

## References

[1] World Health Organization. WHO guidelines for pharmacological management of influenza. Revised February 2010. Part 1: recommendations [cited 2010 Dec 10]. http://www.who.int/csr/resources/publications/swineflu/h1n1_guidelines_pharmaceutical_mngt.pdf

[2] Weingarten S, Friedlander M, Rascon D, Ault M, Morgan M, Meyer RD. Influenza surveillance in an acute-care hospital. Arch Intern Med. 1988;148:113–6. 10.1001/archinte.1988.003800101170113337588

[3] Jacomo V, Sartor C, Zandotti C, Atlan-Gepner C, Drancourt M. Nosocomial influenza outbreak in an intensive-care unit. Med Mal Infect. 2001;31:563–8. 10.1016/S0399-077X(01)00275-X

[4] Rivera M, Gonzalez N. An influenza outbreak in a hospital. Am J Nurs. 1982;82:1836–8.6924541

[5] Horcajada JP, Pumarola T, Martinez JA, Tapias G, Bayas JM, de la Prada M, A nosocomial outbreak of influenza during a period without influenza epidemic activity. Eur Respir J. 2003;21:303–7. 10.1183/09031936.03.0004050312608445

[6] Malavaud S. Nosocomial outbreak of influenza virus A (H3N2) infection in a solid organ transplant department. Transplantation. 2001;72:535–7. 10.1097/00007890-200108150-0003211502991

[7] Hall CB, Douglas RG. Nosocomial influenza infection as a cause of intercurrent fevers in infants. Pediatrics. 1975;55:673–7.1168894

[8] Lester-Smith D, Zurynski YA, Booy R, Festa MS, Kesson AM, Elliott EJ. The burden of childhood influenza in a tertiary paediatric setting. Commun Dis Intell. 2009;33:209–15.1987754010.33321/cdi.2009.33.21

[9] Meibalane R. Outbreak of influenza in a neonatal intensive care unit. J Pediatr. 1977;91:974–6. 10.1016/S0022-3476(77)80907-4925833

[10] Strausbaugh LJ, Sukumar SR, Joseph CL. Infectious disease outbreaks in nursing homes: an unappreciated hazard for frail elderly persons. Clin Infect Dis. 2003;36:870–6. 10.1086/36819712652388

[11] Nuorti JP, Butler JC, Crutcher JM, Guevara R, Welch D, Holder P, An outbreak of multi-drug resistant pneumococcal pneumonia and bacteremia among unvaccinated nursing home residents. N Engl J Med. 1998;338:1861–8. 10.1056/NEJM1998062533826019637804

[12] Yassi A, McGill M, Holton D, Nicolle L. Morbidity, cost and role of healthcare worker transmission in an influenza outbreak in a tertiary care hospital. Can J Infect Dis Med Microbiol. 1993;4:52–6.10.1155/1993/498236PMC325081322346421

[13] Pachucki CT. Influenza A among hospital personnel and patients: implications for recognition, prevention and control. Arch Intern Med. 1989;149:77–80. 10.1001/archinte.1989.003900100910102912418

[14] Catalyud L. Six C, Duponchel J, Sillam F, Charlet F, Leussier J, et al. Épisodes de grippe dans deux établissements d’hébergement pour personnes âgées dans les Bouches-du-Rhône, France, mars-avril 2008. Bulletin épidémiologique hebdomadaire. 2009;18–19:189–192.

[15] Elder AG, O’Donnell B, McCruden EAB, Symington IS, Carman WF. Incidence and recall of influenza in a cohort of Glasgow healthcare workers during the 1993–4 epidemic: results of serum testing and questionnaire. BMJ. 1996;313:1241–2. 10.1136/bmj.313.7067.12418939114PMC2352563

[16] Wilde JA, McMillan JA, Serwint J, Butta J, O’Riordan MA, Steinhoff MC. Effectiveness of influenza vaccine in healthcare professionals. JAMA. 1999;281:908–13. 10.1001/jama.281.10.90810078487

[17] Centers for Disease Control and Prevention. Prevention and control of influenza with vaccines: recommendations of the Advisory Committee on Immunization Practices (ACIP). MMWR Morb Mortal Wkly Rep. 2010;59:1–5.20075837

[18] Burls A, Jordan R, Barton P, Olowokure B, Wake B, Albon E, Vaccinating healthcare workers against influenza to protect the vulnerable—is it a good use of healthcare resources? A systematic review of the evidence and an economic evaluation. Vaccine. 2006;24:4212–21. 10.1016/j.vaccine.2005.12.04316546308

[19] Thomas RE, Jefferson T, Lasserson TJ. Influenza vaccination for healthcare workers who work with the elderly. Cochrane Database Syst Rev. 2010;(2):CD005187.2016607310.1002/14651858.CD005187.pub3

[20] Higgins JPT, Sally Green S. Cochrane handbook for systematic reviews of interventions. Version 5.1.0 [cited 2010 Dec 10]. http://www.mrc-bsu.cam.ac.uk/cochrane/handbook/

[21] Downs SH, Black N. The feasibility of creating a checklist for the assessment of the methodological quality both of randomised and non-randomised studies of healthcare interventions. J Epidemiol Community Health. 1998;52:377–84. 10.1136/jech.52.6.3779764259PMC1756728

[22] West S, King V, Carey T, Lohr K, McKoy N, Sutton S, Systems to rate the strength of scientific evidence. Evidence report/technology appraisal number 47. Rockville (MD): Agency for Healthcare Research and Quality; 2002 [cited 2012 May 30]. http://www.thecre.com/pdf/ahrq-system-strength.pdfPMC478159111979732

[23] Centre for Reviews and Dissemination. Systematic reviews. CRD’s guidance for undertaking reviews in healthcare. York (UK): The Centre, University of York; 2008.

[24] Carman WF, Elder AG, Wallace LA, McAulay K, Walker A, Murray GD, Effects of influenza vaccination of health-care workers on mortality of elderly people in long-term care: a randomised controlled trial. Lancet. 2000;355:93–7. 10.1016/S0140-6736(99)05190-910675165

[25] Potter J, Stott DJ, Roberts MA, Elder AG, O’Donnell B, Knight PV, Influenza vaccination of healthcare workers in long-term-care hospitals reduces the mortality of elderly patients. J Infect Dis. 1997;175:1–6. 10.1093/infdis/175.1.18985189PMC7109672

[26] Lemaitre M, Meret T, Rothan-Tondeur M, Belmin J, Lejonc J, Luquel L, Effect of influenza vaccination of nursing home staff on mortality of residents: a cluster-randomized trial. J Am Geriatr Soc. 2009;57:1580–6. 10.1111/j.1532-5415.2009.02402.x19682118

[27] Hayward AC, Harling R, Wetten S, Johnson AM, Munro S, Smedley J, Effectiveness of an influenza vaccine programme for care home staff to prevent death, morbidity, and health service use among residents: cluster randomised controlled trial. BMJ. 2006;333:1241–2. 10.1136/bmj.39010.581354.5517142257PMC1702427

[28] Oshitani H, Saito R, Seki N, Tanabe N, Yamazaki O, Hayashi S, Influenza vaccination levels and influenza like illness in long term care facilities for elderly people during an Influenza A (H3N2) epidemic. Infect Control Hosp Epidemiol. 2000;21:728–30. 10.1086/50172511089658

[29] Kanaoka S. Inpatient and personnel vaccination influence on influenza outbreaks in long-term medical and care hospital [in Japanese]. Kansenshogaku Zasshi. 2010;84:14–8.20170008

[30] Ando R, Kaname S, Yoshida M, Murakami A, Kurimoto Y, Higaki M, Survey of novel influenza A (H1N1) infection and vaccination in dialysis facilities in the Tokyo Tama area [in Japanese]. Nihon Toseki Igakki Zasshi. 2010;43:891–7.

[31] Engels O, Goldman N, Doyen M, Duyse M, Van Beers D, Vergison A. Reduction of the nosocomial influenza A burden in a paediatric hospital by immunisation of the healthcare workers. Abstract no. 1133_242. Presentation at the 15th European Congress of Clinical Microbiology and Infectious Diseases; 2005 April 2–5; Copenhagen, Denmark.

[32] Weinstock DM, Eagan J, Malak SA, Rogers M, Wallace H, Kiehn TE, Control of influenza A on a bone marrow transplant unit. Infect Control Hosp Epidemiol. 2000;21:730–2. 10.1086/50172611089659

[33] Saito R, Suzuki H, Oshitani H, Sakai T, Seki N, Tanabe N. The effectiveness of influenza vaccine against influenza a (H3N2) virus infections in nursing homes in Niigata, Japan, during the 1998–1999 and 1999–2000 seasons. Infect Control Hosp Epidemiol. 2002;23:82–6. 10.1086/50201111893153

[34] Munford C, Finnigan S. Influenza campaign 2006 and 2007: a residential care success story. Can J Infect Control. 2008;23:222–5, 227.19350999

[35] Shugarman LR, Hales C, Setodji CM, Bardenheier B, Lynn J. The influence of staff and resident immunization rates on influenza-like illness outbreaks in nursing homes. J Am Med Dir Assoc. 2006;7:562–7. 10.1016/j.jamda.2006.06.00217095421

[36] Monto AS, Rotthoff J, Teich E, Herlocher ML, Truscon R, Yen H, Detection and control of influenza outbreaks in well-vaccinated nursing home populations. Clin Infect Dis. 2004;39:459–64. 10.1086/42264615356805

[37] Stevenson CG, McArthur MA, Naus M, Abraham E, McGeer AJ. Prevention of influenza and pneumococcal pneumonia in Canadian long-term care facilities: how are we doing? CMAJ. 2001;164:1413–9.11387913PMC81067

[38] Talbot TR, Babcock H, Caplan AL, Cotton D, Maragakis LL, Poland GA, Revised SHEA position paper: influenza vaccination of healthcare personnel. Infect Control Hosp Epidemiol. 2010;31:987–95. 10.1086/65655820807037

[39] van den Dool C, Bonten MJ, Hak E, Wallinga J. Modeling the effects of influenza vaccination of healthcare workers in hospital departments. Vaccine. 2009;27:6261–7. 10.1016/j.vaccine.2009.07.10419686690

[40] Anikeeva O, Braunack-Mayer A, Rogers W. Requiring influenza vaccination for healthcare workers. Am J Public Health. 2009;99:24–9. 10.2105/AJPH.2008.13644019008501PMC2636609

# Review article: Vaccination of Health Care Workers to Protect Patients at Increased Risk for Acute Respiratory Disease

**DOI:** 10.3201/eid1808.111355

**Authors:** Gayle P. Dolan, Rebecca C. Harris, Mandy Clarkson, Rachel Sokal, Gemma Morgan, Mitsuru Mukaigawara, Hiroshi Horiuchi, Rachel Hale, Laura Stormont, Laura Béchard-Evans, Yi-Sheng Chao, Sergey Eremin, Sara Martins, John S. Tam, Javier Peñalver, Arina Zanuzadana, Jonathan S. Nguyen-Van-Tam

**Affiliations:** University of Nottingham, Nottingham, UK (G.P. Dolan, R. Hale, J.S. Nguyen-Van-Tam);; World Health Organization, Geneva, Switzerland (R.C. Harris, M. Mukaigawara, L. Stormont, Laura Béchard-Evans, Y.-S. Chao, S. Eremin, S. Martins, J.S. Tam, J. Peñalver);; National Health Service Derbyshire County, Chesterfield, UK (M. Clarkson, R. Sokal);; Health Protection Agency South West, Gloucester, UK (G. Morgan);; Tokyo Medical Dental University, Tokyo, Japan (H. Horiuchi);; and University of Bielefeld, Bielefeld, Germany (A. Zanuzadana)

## Medscape CME Quiz

## Earning CME Credit

To obtain credit, you should first read the journal article. After reading the article, you should be able to answer the following, related, multiple-choice questions. To complete the questions (with a minimum 70% passing score) and earn continuing medical education (CME) credit, please go to www.medscape.org/journal/eid. Credit cannot be obtained for tests completed on paper, although you may use the worksheet below to keep a record of your answers. You must be a registered user on Medscape.org. If you are not registered on Medscape.org, please click on the New Users: Free Registration link on the left hand side of the website to register. Only one answer is correct for each question. Once you successfully answer all post-test questions you will be able to view and/or print your certificate. For questions regarding the content of this activity, contact the accredited provider, CME@medscape.net. For technical assistance, contact CME@webmd.net. American Medical Association’s Physician’s Recognition Award (AMA PRA) credits are accepted in the US as evidence of participation in CME activities. For further information on this award, please refer to http://www.ama-assn.org/ama/pub/category/2922.html. The AMA has determined that physicians not licensed in the US who participate in this CME activity are eligible for *AMA PRA Category 1 Credits*™. Through agreements that the AMA has made with agencies in some countries, AMA PRA credit may be acceptable as evidence of participation in CME activities. If you are not licensed in the US, please complete the questions online, print the certificate and present it to your national medical association for review.

## Article Title: Vaccination of Health Care Workers to Protect Patients at Increased Risk for Acute Respiratory Disease

## CME Questions

**1. Several employees in your health care system are complaining about a policy of universal influenza vaccination announced several years ago. They feel that mandatory vaccinations (with substantial precautions for workers who opt out of vaccinations) are unfair and may not be effective.**

**You need to craft an answer to their complaints. What should you consider regarding influenza infection among health care workers?**

A. More than half of health care workers have clinical infection with influenza during a given season

B. Nearly all young adults infected with influenza will have clinical symptoms of infection

C. Influenza vaccination has not been effective in protecting against influenza B strains

D. Universal influenza vaccination of health care workers is likely to be cost-effective

**2. Regarding the current systematic review by Dolan and colleagues, what should you consider regarding the methods of research into vaccination of health care workers and patient protection from illness?**

A. Vaccination rates among staff in the intervention groups were nearly 100% across all studies

B. Most research was conducted in long-term care facilities

C. Vaccine coverage among patients was limited between 5% and 20%

D. All studies demonstrated good blinding of participants and study personnel

**3. Based on the current systematic review, what can you tell your colleagues regarding the effects of influenza vaccination for health care workers on patient cases and consultations for ILI (ILI)?**

A. There is no effect on health care worker vaccination on patients’ risk of ILI or consultation for ILI

B. Vaccination of health care workers reduces patients’ risk of ILI but not rates of consultation for ILI

C. Vaccination of health care workers reduces patients’ rates of consultation for ILI but not ILI itself

D. Vaccination of health care workers reduces patients’ risk of ILI as well as rates of consultation for ILI

**4. Which of the following outcomes among patients appears to be most improved with influenza vaccination of health care workers?**

A. All-cause mortality

B. Death due to respiratory causes

C. Death due to pneumonia

D. Hospitalization for respiratory causes

## Activity Evaluation

**Table:** 

**1. The activity supported the learning objectives.**				
Strongly Disagree				Strongly Agree
1	2	3	4	5
**2. The material was organized clearly for learning to occur.**				
Strongly Disagree				Strongly Agree
1	2	3	4	5
**3. The content learned from this activity will impact my practice.**				
Strongly Disagree				Strongly Agree
1	2	3	4	5
**4. The activity was presented objectively and free of commercial bias.**				
Strongly Disagree				Strongly Agree
1	2	3	4	5
